# The impact of exercise on blood-based biomarkers of Alzheimer’s disease in cognitively unimpaired older adults

**DOI:** 10.1007/s11357-024-01130-2

**Published:** 2024-03-15

**Authors:** Kelsey R. Sewell, Stephanie R. Rainey-Smith, Steve Pedrini, Jeremiah J. Peiffer, Hamid R. Sohrabi, Kevin Taddei, Shaun J. Markovic, Ralph N. Martins, Belinda M. Brown

**Affiliations:** 1 Centre for Healthy Ageing, Health Futures Institute, Murdoch University, Murdoch, WA Australia; 2 School of Medical and Health Sciences, Edith Cowan University, Joondalup, WA Australia; 3 Alzheimer’s Research Australia, Ralph and Patricia Sarich Neuroscience Research Institute, Nedlands, WA Australia; 4 School of Psychological Science, University of Western Australia, Crawley, WA Australia; 5 Department of Biomedical Sciences, Macquarie University, North Ryde, New South Wales Australia

**Keywords:** Blood biomarkers, Exercise, Alzheimer’s disease, Cognition

## Abstract

Physical activity is a promising preventative strategy for Alzheimer’s disease: it is associated with lower dementia risk, better cognition, greater brain volume and lower brain beta-amyloid. Blood-based biomarkers have emerged as a low-cost, non-invasive strategy for detecting preclinical Alzheimer’s disease, however, there is limited literature examining the effect of exercise (a structured form of physical activity) on blood-based biomarkers. The current study investigated the influence of a 6-month exercise intervention on levels of plasma beta-amyloid (Aβ42_,_ Aβ40, Aβ42/40), phosphorylated tau (p-tau181), glial fibrillary acidic protein (GFAP) and neurofilament light (NfL) chain in cognitively unimpaired older adults, and as a secondary aim, whether blood-based biomarkers related to cognition. Ninety-nine community-dwelling older adults (69.1 ± 5.2) were allocated to an inactive control, or to moderate or high intensity exercise groups where they cycled twice weekly for six months. At baseline and six months (post-intervention), fasted blood was collected and analysed using single molecule array (SIMOA) assays, and cognition was assessed. Results demonstrated no change in levels of any plasma biomarker from pre- to post-intervention. At baseline, higher NfL was associated with poorer cognition (*β* = -0.33, *SE* = 0.13, adjusted *p* = .042). Exploratory analyses indicated higher cardiorespiratory fitness was associated with higher NfL and GFAP levels in apolipoprotein E (*APOE*) ε4 non-carriers compared to ε4 carriers (NfL, *β* = -0.43, *SE* = 0.19, *p* = .029; GFAP, *β* = -0.41, *SE* = 0.20, *p* = .044), though this association was mediated by body mass index (BMI). These results highlight the importance of considering BMI in analysis of blood-based biomarkers, especially when investigating differences between *APOE* ε4 carriers and non-carriers. Our results also indicate that longer follow-up periods may be required to observe exercise-induced change in blood-based biomarkers.

**Supplementary information:**

The online version contains supplementary material available at 10.1007/s11357-024-01130-2.

## Introduction

Physical activity is associated with reduced risk of Alzheimer’s disease (AD) [1], and may be best utilised prior to the onset of symptoms (i.e., in cognitively unimpaired individuals), before neuronal damage becomes irreversible [2]. Exercise interventions (a structured form of physical activity) can improve cognition and brain structure in healthy older adults [3]. Moreover, exercise may reduce levels of beta-amyloid (Aβ) and tau, pathological hallmarks of AD, in both human and animal models [4]. The presence of brain Aβ and tau, along with other disease biomarkers for astrogliosis and neurodegeneration, may be reflected in the periphery [5], measured through blood serum and plasma. Blood-based biomarkers present a non-invasive, low-cost methodology of examining early AD risk, and are more attractive as large-scale screening and diagnostic tools, compared to costly imaging and invasive collection of cerebrospinal fluid (CSF). Importantly, abnormalities in blood and CSF biomarkers may occur before brain imaging changes are visible [6]. Thus, it is crucial to examine how interventions for AD prevention, such as exercise, may influence AD-related blood-based biomarkers before cognitive symptoms manifest.

There have been marked advances in the reliability, specificity and sensitivity, of blood-based biomarkers for AD [5, 7]. The plasma Aβ42/40 ratio reflects levels of brain Aβ with high accuracy, with area under the curve ranging from 0.80 – 0.90, overall accuracy from 75% – 81%, and accuracy up to 90% for a composite plasma biomarker [7–10]. A biomarker panel may be most useful for differentiating AD stages and predicting cognitive decline [7, 11]. In particular, Aβ42_,_ Aβ40_,_ p-tau181, glial fibrillary acidic protein (GFAP; an indicator of astrogliosis) and neurofilament light (NfL; a marker of axonal injury) show reliability for reflecting neuropathological processes and predicting AD development [5, 12]. Plasma levels of GFAP, NfL, and p-tau181 are increased in mild cognitive impairment (which often precedes AD) and AD, compared to cognitively unimpaired individuals [13]. By contrast, plasma Aβ42 and Aβ40 levels are lowered in AD [14]. In cognitively unimpaired individuals, higher plasma NfL and GFAP have been associated with worse cognitive performance [14, 15], and lower Aβ42/40 ratio, higher p-tau181, GFAP and NfL are associated with more rapid cognitive decline [14, 16].

Many studies have examined the association between exercise and AD-related outcomes such as cognition and brain structure [3], however few studies have examined how exercise influences blood-based AD biomarkers. This may be owing to the lack of specificity and sensitivity of plasma biomarkers to detect abnormal biomarker levels prior to recent technological advances, such as use of the ultrasensitive single molecule array (SIMOA) [9], which has demonstrated improvements in accuracy for reflecting brain Aβ levels compared to previous technologies [7, 17]. Exercise intervention studies conducted before the introduction of SIMOA show a decrease in Aβ42 and Aβ42/40 in cognitively unimpaired individuals in exercise groups compared to controls [18, 19]. Conversely, one recent observational study showed no association between physical activity and blood Aβ42/40 [20], however, this association may depend on apolipoprotein E (*APOE*) ε4 status [21, 22], and the interaction with *APOE* ε4 was not examined in this instance. Although recent studies have investigated the effects of exercise on blood-based biomarkers in AD patients [23, 24], there is a lack of published studies which investigate the impact of exercise interventions on blood-based biomarkers (measured using SIMOA technology) in cognitively unimpaired individuals, which is important for determining the effectiveness of exercise to delay or prevent the onset of AD.

Given recent advances in the accuracy of blood biomarker detection of AD, and evidence that exercise is a protective factor against AD risk [2], it is important to examine the effect of exercise on blood-based biomarkers in cognitively unimpaired older adults. The current study was a secondary analysis of data from the Intense Physical Activity and Cognition (IPAC) study, a 6-month randomised controlled trial using exercise to improve cognition in cognitively unimpaired older adults [25]. Our primary aim was to examine whether exercise influenced levels of plasma Aβ42_,_ Aβ40_,_ Aβ42/40 ratio, p-tau181, GFAP and NfL from pre- to post-intervention. We also investigated baseline associations between cardiorespiratory fitness, cognition, and plasma biomarkers. As secondary aims, we examined whether baseline associations varied by *APOE* ε4 carriage status, and whether any changes in biomarkers across the intervention were related to changes in cognition. Finally, as an exploratory post-hoc analysis, we examined whether cardiometabolic factors (specifically body mass index; BMI) influenced associations between cardiorespiratory fitness and plasma biomarkers.

## Methods

### Participants

**Fig. 1:**
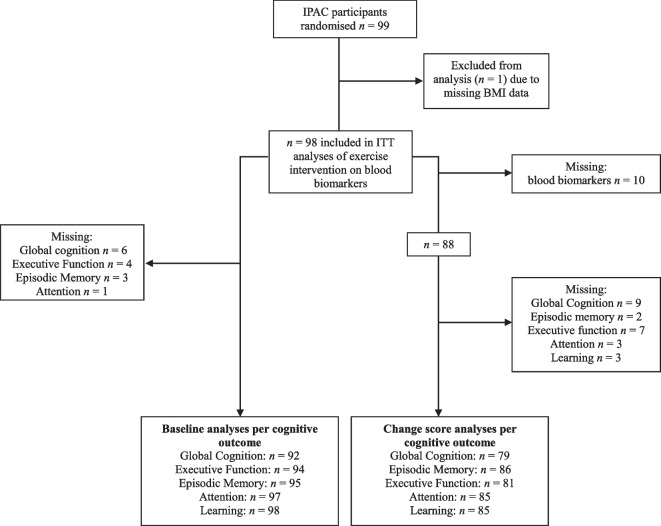
Flow diagram indicating number of participants with data available for inclusion for each analysis. The complete Consolidated Standards Of Reporting Trials diagram is detailed in Brown et al. [26]. Abbreviations: IPAC, Intense Physical Activity and Cognition study; BMI, body mass index; ITT, intention-to-treat

Ninety-nine cognitively unimpaired community-dwelling older adults (aged 60–80) participated in the Intense Physical Activity and Cognition (IPAC) study. The primary endpoint of the IPAC study was to evaluate the impact of exercise on cognitive function and these results have been published previously in Brown et al. 2021 [26]. For the current intention-to-treat analyses, the sample size was *n* = 98 because one person had missing BMI data. In baseline analyses involving cognition, sample size varied from *n* = 98 (learning) to *n* = 92 (global cognition) due to missing data. For change score analyses involving biomarkers and cognition from pre- to post-intervention, the total sample size was *n* = 86 (learning) varying to *n* = 75 (global cognition); sample size is fully detailed in Fig. 1. Full inclusion and exclusion criteria have been described previously [25], but briefly, participants were excluded if they: scored ≤ 26 on the Mini–Mental State Examination, scored ≥ 6 on the Geriatric Depression Scale, had diabetes, uncontrolled hypertension, untreated obstructive sleep apnea, history of bipolar, schizophrenia or schizoaffective disorders. Data were collected from 1st February, 2017 to 25th September, 2019. A block randomisation protocol was used to allocate participants into either 6-months of high intensity exercise, moderate intensity exercise, or an inactive control group. See Brown et al. [26] for the Consolidated Standards Of Reporting Trials (CONSORT) diagram and for additional participant information.

The IPAC study is registered with the Australian New Zealand Clinical Trials Registry (ACTRN12617000643370). The human research ethics committees at Murdoch University and Edith Cowan University approved the conduct of this study, and individuals provided written informed consent prior to participation.

### Intervention protocol

Participants in the exercise groups (i.e., moderate intensity and high intensity groups) cycled on an ergometer (Wattbike Pro; Wattbike, Australia) for 50 min, twice a week for six months. Sessions were completed in a university lab setting supervised by an exercise physiologist, with a maximum of three participants to one researcher in each session. Adherence was measured via session attendance logs, and target exercise intensity set and maintained via rating of perceived exertion, allowing participants to maintain a relative level of intensity consistent with changes in fitness (see [26] for additional information). Those in the high intensity exercise group completed a 10-min warm up, 11 intervals of one minute cycling at hard exertion at 18.0 Borg Scale, 80% aerobic capacity (VO_2peak_, see ‘fitness assessment’ below), interspersed with two minutes of active recovery, and a 9-min cool down. The moderate intensity group cycled at a constant intensity for 50 min (50–60% aerobic capacity; 13.0 Borg Scale). The control group received one, 2-h information session regarding the benefits of exercise to overall health and the brain.

### Cardiorespiratory fitness and physical activity assessment

At baseline, 3, and 6 months, participants underwent a cycling-based graded exercise test to quantify peak aerobic capacity (VO_2peak_) and peak power. All tests used 2-min stage durations with consistent increases in work rate at each stage, based on participant body mass, until participants reached volitional fatigue. During the test, heart rate and ventilatory gases were continuously recorded and averaged into 15-s intervals by a metabolic cart (TrueOne 2400, Parvomedics, Salt Lake City, USA). VO_2peak_ was defined as the greatest consecutive 15-s mean values during the final two minutes of the test. For a full description of the fitness assessment see Brown et al. [25]. Baseline physical activity levels were evaluated using the International Physical Activity Questionnaire (IPAQ) [27] and were only used to characterise the sample (Table 1).

**Table 1:** Descriptive statistics for the IPAC cohort

	High Intensity (*n* = 33)	Moderate Intensity (*n* = 34)	Control (*n* = 32)	Test Statistic
Age, years	70.2 (5.3)	68.4 (4.2)	68.7 (5.9)	*F* = 1.22
Gender, % Female (n)	51.5 (17)	52.9 (18)	59.4 (19)	*χ*^*2*^ = 0.79
*APOE* ε4 allele carriers, % (n)	27.3 (9)	23.5 (8)	28.1 (9)	*χ*^*2*^ = 0.90
Years of education	13.5 (2.2)	14.2 (2.5)	14.5 (2.1)	*F* = 1.65
Global cognition, MoCA	26.0 (2.1)	26.4 (2.8)	26.7 (2.0)	*F* = 0.64
Alcohol, Units per week	5.7 (5.9)	5.1 (5.5)	6.4 (6.1)	*F* = 0.44
Depression score, DASS	2.3 (3.0)	1.6 (2.1)	1.7 (1.9)	*F* = 0.95
Body Mass Index, kg/m^2^	25.8 (3.7)	26.0 (3.9)	25.3 (3.4)	*F* = 0.30
Physical Activity, (MET min/week^−1^)	4379 (3708)	4372 (2488)	3533 (1981)	*F* = 0.94
Baseline VO_2peak_, mL/kg/min	22.2 (6.3)	24.7 (6.9)	22.8 (6.1)	*F* = 1.36
Plasma Aβ40, pg/mL	87.2 (14.7)	86.7 (15.7)	86.4 (13.7)	*F* = 0.02
Plasma Aβ42, pg/mL	5.3 (1.2)	5.1 (1.7)	5.6 (1.1)	*F* = 0.28
Plasma Aβ42/40	0.06 (0.01)	0.06 (0.02)	0.07 (0.01)	*F* = 2.80
Plasma GFAP, pg/mL	129.5 (76.1)	120.2 (63.6)	118.6 (39.7)	*F* = 0.30
Plasma NfL, pg/mL	17.8 (10.0)	16.9 (6.6)	17.6 (5.9)	*F* = 0.13
Plasma p-tau181, pg/mL	2.2 (1.0)	2.2 (0.9)	2.1 (0.8)	*F* = 0.25

Unless otherwise described, data are presented as mean (standard deviation). Differences between groups were calculated using analysis of variance for continuous variables and chi-square for categorial variables. Abbreviations: *IPAC* Intense Physical Activity and Cognition, *APOE* Apolipoprotein E gene, *MoCA* Montreal Cognitive Assessment, *DASS* Depression Anxiety and Stress Scale, *kg/m*^*2*^ kilograms per metre squared, *MET* metabolic task equivalent, *VO*_*2peak*_ volume of oxygen uptake during peak exercise, *Aβ* amyloid-beta, *pg/mL* picograms per millilitre, *GFAP* Glial Fibrillar Acidic Protein *NfL* Neurofilament Light chain, *p-tau181* phosphorylated tau 181

^*^p < .05, ** p < *.001*

### Blood-based biomarkers

Levels of p-tau181, Aβ40, Aβ42, GFAP, and NfL were measured in fasted plasma EDTA samples using the SIMOA platform (Quanterix, Billerica, MA, USA). Samples aimed to be collected at the same time for each participant, always in the morning and usually at approximately 8am. Aβ40, Aβ42, GFAP, and NfL were measured using the Neurology 4-Plex E assay (QTX-103670, Quanterix, Billerica, MA, USA). Calibrators were run in duplicate while internal controls and samples were run in singlicate. Average Coefficient of Variation (CV) % of previous batches run in duplicate in our laboratory for Aβ40, Aβ42, GFAP, and NfL were 1.56%, 2.91%, 3.26%, and 3.20%, respectively. Levels of p-tau181 were measured using the p-tau181 V2.0 SIMOA Advantage Assay (QTX-103714, Quanterix, Billerica, MA, USA). Calibrators, internal controls, and samples were run in duplicate. Average CV% for p-tau181 in plasma samples was 7.58%. The analytical lowest limit of quantification of our analytes was 4.08 pg/mL for Aβ40, 1.51 pg/mL for Aβ42, 11.6 pg/mL for GFAP, 1.6 pg/mL for NfL and 0.338 pg/mL for p-tau181, as indicated in the manufacturer’s instructions. Quality control of our analyses was confirmed by determining that the levels of the internal controls included in the SIMOA assays were within the range provided by the manufacturer.

### Cognitive assessment

A cognitive assessment was administered to all participants at baseline and 6 months. The cognitive battery included the California Verbal Learning Test second edition (CVLT-II), Montreal Cognitive Assessment (MoCA), Brief Visual Memory Test (BVMT), Wechsler Adult Intelligence Scale-III Digit Span, Trail Making Tests A and B, the unstructured task, verbal fluency, flanker, and set-shifting from the from the National Institutes of Health—Executive Abilities: Measures and Instruments for Neurobehavioral Evaluation and Research (NIH-EXAMINER), and The Cogstate battery, a computerised cognitive assessment (www.cogstate.com). We calculated composite scores for global cognition, attention, episodic memory, and executive function based on previously published and validated composites [25] (see Supplementary Table 1 for tests included in each composite). *Z*-scores were calculated using individual’s performance across all timepoints. For tasks where a lower score indicates better functioning (i.e., reaction time), the score was inversed ([score]*-1). Composite scores were then created by calculating the mean performance across the respective tasks for each composite (see Supplementary Table 1).

### Statistical analyses

All analyses were conducted using R statistical computing packages version 4.2.1. To compare demographics across control, moderate intensity and high intensity exercise groups, analyses of variance were conducted for continuous variables, and chi-square tests were conducted for categorical variables. For all analyses which examined change from pre- to post-intervention, intention-to-treat (ITT) analyses were performed, such that all participants with complete baseline data were included, regardless of study withdrawal. Outliers for biomarker variables (*n* = 1 for all analytes except Aβ42 where* n* = 2) were determined as those greater than ± 3 standard deviations from the mean and were Winsorised (i.e., replaced with the next closest value which was not an outlier). A sensitivity analysis indicated only results for change in biomarkers and change in cognition from pre- to post-intervention differed due to winsorisation; both winsorised and non-winsorised results are reported below. The false discovery rate (FDR) correction [28] for multiple comparisons was applied for all analyses except those which were exploratory [29]; FDR corrected *p*-values are reported as *q*-values.

We used linear mixed models (LMMs) to investigate our primary aim of whether exercise influenced biomarker change from pre- to post-intervention. The respective biomarker was entered as the dependent variable, the time*group interaction entered as fixed factors, and participant ID as a random factor. We also used LMMs to determine whether any effects of exercise on biomarkers varied due to *APOE* ε4 carriage status (time*group*APOE), with *APOE* genotype determined using TaqMan genotyping assays per standard protocols [30], resulting in a dichotomous variable for *APOE* ε4 carriers and non-carriers. Finally, we calculated change scores (post level/score – pre level/score) for each biomarker, cognitive domain, and fitness, and conducted linear models to determine whether change in biomarkers was associated with change in cognition (regardless of intervention group), and whether change in fitness was associated with changes in biomarkers (regardless of intervention group). All models included age, sex, BMI and *APOE* ε4 carriage status (when not included as an interaction term) as covariates.

We used linear models to characterise baseline (cross-sectional) associations between biomarker levels (predictor variable) and cognition (dependent variable), with separate models for each biomarker on each cognitive domain. As a retrospective exploratory analysis, we used linear models to examine baseline associations between fitness (predictor variable) and biomarker levels (dependent variable). The PROCESS macro for R was used to conduct an exploratory, retrospective mediation to better understand unexpected baseline associations (see Results below). For all regression analyses, variables were mean-centred where appropriate (i.e., for all continuous variables except time), but left as raw data in graphs for ease of interpretation.

## Results

Descriptive data for the IPAC cohort, separated by exercise group, are presented in Table 1. The full sample had a mean age of 69.1 ± 5.2 years and were 55% female; there were no differences between exercise intervention groups for any variable (Table 1).

### Influence of exercise on plasma biomarkers from pre- to post-intervention by *APOE* ε4 carriage status

Collapsed across intervention groups, there were no changes in any of the investigated plasma biomarkers from pre- to post-intervention (Table 2). There were no significant time*group interactions for change in biomarkers from pre- to post-intervention, and this did not differ between *APOE* ε4 carriers and non-carriers (Table 2).

**Table 2:** Linear mixed models examining change in plasma biomarkers and cardiorespiratory fitness from pre- to post-exercise intervention by group and *APOE* ε4 carriage status

Raw mean change from baseline [95% CI]			Time (ITT) Unstandardized B (standard error) *Model 1*	Time*Group (ITT) Unstandardized B (standard error) *Model 2*	Time*Group*APOE (ITT) Unstandardized B (standard error) *Model 3*
Control (*n* = 27)	Moderate-intensity (*n* = 31)	High-intensity (*n* = 30)			
**VO**_**2**_**peak (ml/kg/min)**			4.43 (0.64)***	3.71(0.70)***	1.77 (1.53)
-0.76 [-3.6, 2.1]	3.09 [-0.4, 6.5]	5.60 [2.4, 8.7]			
**Plasma Aβ40**			1.03 (1.70)	0.62 (2.16)	-4.31 (4.72)
-2.17 [-9.8, 5.4]	2.59 [-4.6, 9.8]	1.59 [-9.3, 12.44]			
**Plasma Aβ42**			0.08 (0.12)	-0.02 (0.16)	0.02 (0.35)
0.07 [-0.6, 0.8]	-0.06 [-0.8, 0.8]	0.11 [-0.7, 0.9]			
**Plasma Aβ42/40**			0.001 (0.001)	-0.001 (0.002)	0.003 (0.003)
0.002 [-0.003, 0.008]	-0.002 [-0.009, 0.006]	0.000 [-0.005, 0.006]			
**Plasma GFAP**			7.25 (3.98)	-5.40 (4.98)	-0.25 (10.75)
1.51 [-22.7, 25.7]	3.93 [-28.3, 36.2]	4.30 [-36.5, 45.0]			
**Plasma NfL**			0.27 (0.47)	0.25 (0.59)	1.96 (1.27)
0.07 [-3.3, 3.2]	-0.03 [-3.4, 3.4]	0.62 [-4.5, 5.7]			
**Plasma p-tau181**			-0.08 (0.09)	0.01 (0.11)	0.40 (0.24)
-0.14 [-0.5, 0.2]	0.04 [-0.5, 0.5]	-0.09 [-0.5, 0.3]			

Covariates for all models include age*sex, *APOE* ε4 carriage status (if not included as an interaction term), and body mass index. Abbreviations: *APOE* apolipoprotein E gene, *CI* Confidence Interval, *ITT* Intention-to-Treat, *VO*_*2peak*_ volume of oxygen uptake during peak exercise, *Aβ* amyloid-beta, *GFAP* Glial Fibrillar Acidic Protein, *NfL* Neurofilament Light chain, *p-tau181* phosphorylated tau 181

^*^ *p* < .05, ** p < .01, *** p < .001

### Change in plasma biomarkers, fitness and cognition from pre- to post-intervention

Change in plasma biomarkers did not predict change in any cognitive outcome from pre- to post-intervention (Supplementary Table 2). Similarly, changes in fitness did not predict changes in any plasma biomarker (Supplementary Table 2).

We conducted a sensitivity analysis to determine whether winsorisation of plasma biomarker variables influenced results. Without winsorisation, increases in Aβ42 and NfL were associated with decreases in learning performance from pre- to post-intervention (*β* = -0.22, *SE* = 0.11, uncorrected *p* = 0.039; *β* = -0.24, *SE* = 0.10, uncorrected *p* = 0.024), and increases in p-tau181 and NfL were associated with decreases in global cognition (*β* = -0.31, *SE* = 0.11, uncorrected *p* = 0.007; *β* = -0.27, *SE* = 0.11, uncorrected *p* = 0.015). However, further exploration indicated these results were likely driven by a single participant whose values were winsorised for each plasma variable and who showed large increases in p-tau181, Aβ and NfL from pre- to post-intervention.

### Cross-sectional associations between plasma biomarkers and cognition

In our cross-sectional analyses of baseline data in the whole sample (i.e., not considering intervention group), higher plasma Aβ40 was associated with poorer attention (*p* = 0.014, *q* = 0.07) and higher plasma Aβ42/40 was associated with better episodic memory (*p* = 0.044, *q* = 0.22; Table 3). Higher plasma NfL was associated with poorer learning (*p* = 0.017, *q* = 0.042), episodic memory (*p* = 0.036, *q* = 0.045), executive function (*p* = 0.029, *q* = 0.045), and global cognition (*p* = 0.012, *q* = 0.042; Table 3), higher plasma GFAP was associated with poorer executive function (*p* = 0.027, *q* = 0.135), higher plasma p-tau181 was associated with poorer attention (*p* = 0.034, *q* = 0.057), executive function (*p* = 0.027, *q* = 0.057), and poorer global cognition (*p* = 0.030, *q* = 0.057; Table 2). However, only associations between plasma NfL and cognitive outcomes remained significant after correction for multiple comparisons (see q-values above).

**Table 3:** Linear regression of baseline associations between biomarkers, cardiorespiratory fitness and cognition

	Attention	Learning	Episodic Memory	Executive Function	Global Cognition	Cardiorespiratory fitness
Plasma Aβ40	-0.27 (0.11)*	0.05 (0.10)	0.02 (0.11)	-0.13 (0.10)	-0.09 (0.10)	-0.13 (0.15)
Plasma Aβ42	-0.16 (0.11)	0.12 (0.10)	0.17 (0.10)	-0.11 (0.11)	-0.04 (0.11)	-0.09 (0.15)
Plasma Aβ42/40	0.00 (0.11)	0.12 (0.10)	0.21 (0.10)*	-0.01 (0.12)	0.05 (0.12)	0.00 (0.15)
Plasma GFAP	-0.11 (0.12)	-0.12 (0.12)	-0.06 (0.12)	-0.26 (0.12)*	-0.19 (0.12)	-0.10 (0.13)
Plasma NfL	-0.11 (0.13)	-0.29 (0.12)*	-0.27 (0.13)*	-0.27 (0.12)*	-0.33 (0.13)*	0.08 (0.13)
Plasma p-tau181	-0.24 (0.11)*	-0.07 (0.11)	-0.19 (0.11)	-0.26 (0.11)*	-0.25 (0.11)*	0.10 (0.14)

Covariates for all models include age*sex, *APOE* ε4 carriage status, and body mass index. Reported as standardised β (standard error). Abbreviations: *Aβ* amyloid-beta, *GFAP* Glial Fibrillar Acidic Protein, *NfL* Neurofilament Light chain, *p-tau181* phosphorylated tau 181

^*^ *p* < .05, ** p < .01, *** p < .001. Only associations between NfL and cognitive outcomes remained after correction for multiple comparisons

### Exploratory analyses of cross-sectional associations between fitness and plasma biomarkers

**Fig. 2:**
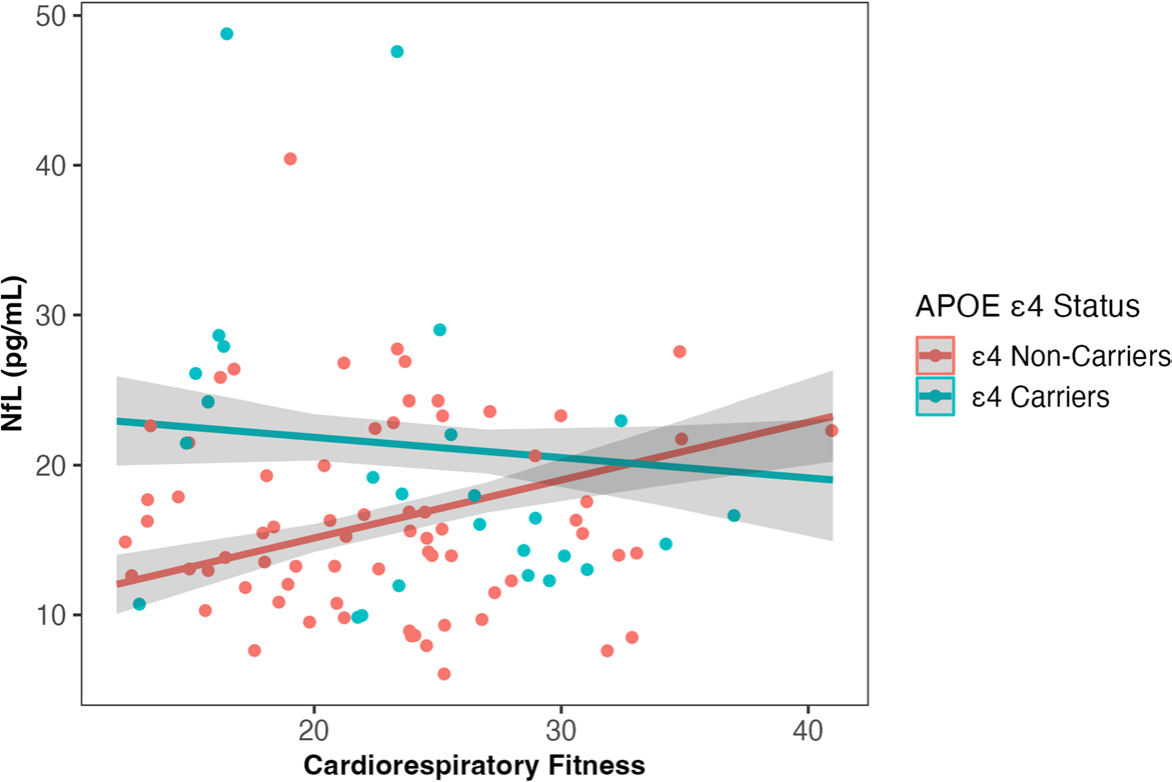
Interaction between cardiorespiratory fitness (measured by VO_2peak_ mL/kg/min) and *APOE* ε4 carrier status on plasma NfL levels at baseline, adjusted for age and sex (*β* = -0.43, *SE* = 0.19, *p* = .029). The slope of fitness on NfL is significant within *APOE* ε4 non-carriers (*p* = .02), but not carriers (*p* = .53). Abbreviations: *APOE*, apolipoprotein E gene; NfL, Neurofilament Light chain; VO_2peak_, volume of oxygen uptake during peak exercise

**Fig. 3:**
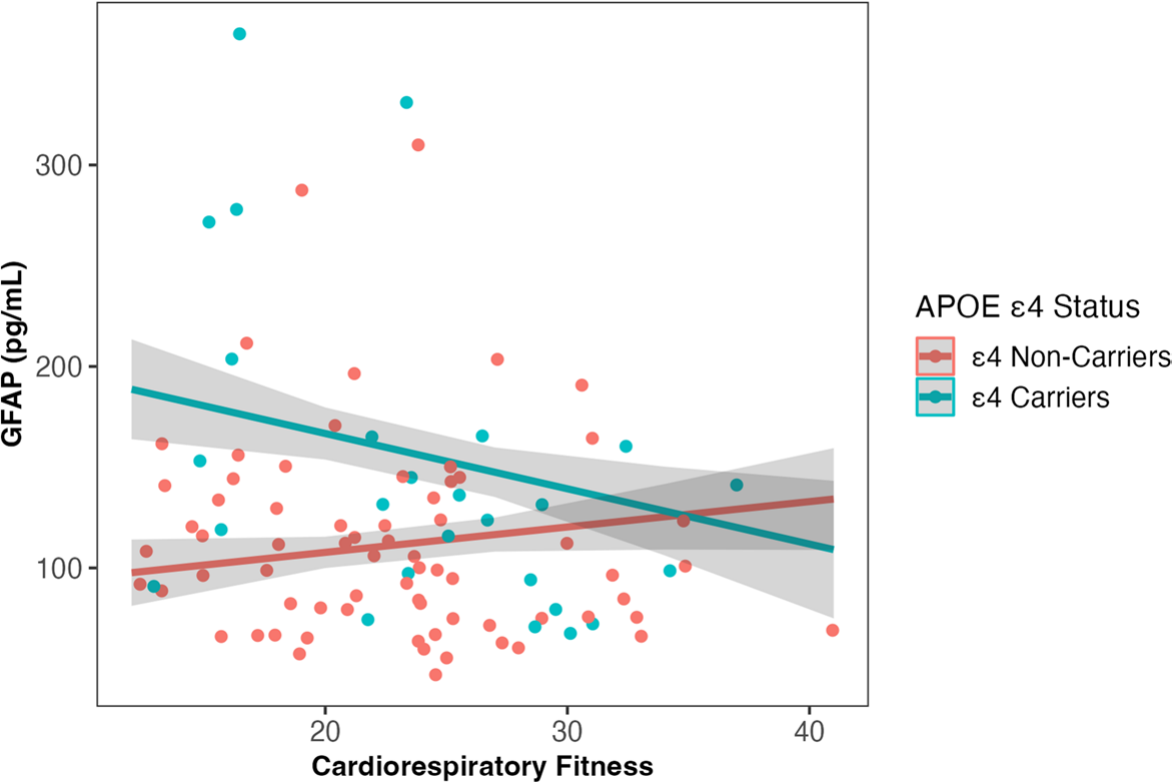
Interaction between cardiorespiratory fitness (measured by VO_2peak_ mL/kg/min) and *APOE* ε4 carrier status on plasma GFAP levels at baseline, adjusted for age and sex (*β* = -0.41, *SE* = 0.20, *p* = .044). The slope of fitness on GFAP is non-significant within *APOE* ε4 carriers (*p* = .15) and non-carriers (*p* = .36). Abbreviations: APOE, apolipoprotein E gene; GFAP, Glial Fibrillary Acidic Protein; VO_2peak_, volume of oxygen uptake during peak exercise

Our initial analyses showed no cross-sectional baseline associations between cardiorespiratory fitness and any of the investigated biomarkers (Table 2). Unadjusted models showed an interaction between *APOE* ε4 carrier status and fitness on both plasma NfL and GFAP (*β* = -0.47, *SE* = 0.22, *p* = 0.034; *β* = -0.43, *SE* = 0.21, *p* = 0.041, respectively). This interaction remained after adjusting for age and sex (NfL, *β* = -0.43, *SE* = 0.19, *p* = 0.029; GFAP, *β* = -0.41, *SE* = 0.20, *p* = 0.044), however became non-significant after adjusting for BMI (NfL, *p* = 0.061; GFAP, *p* = 0.087). In the age- and sex-adjusted model, higher fitness was associated with lower plasma GFAP and NfL in ε4 carriers, however in ε4 non-carriers, higher fitness was associated with higher levels of plasma GFAP and NfL (Figs. 2 and 3).

We conducted exploratory analyses to better understand our unexpected finding that higher fitness was associated with *higher* plasma GFAP and NfL in *APOE* ε4 non-carriers. A t-test indicated that *APOE* ε4 non-carriers had higher BMI (*M* = 26.38, *SD* = 3.7) than carriers (*M* = 23.80, *SD* = 3.0; *p* < 0.001), and linear regression indicated *APOE* ε4 carriage predicted BMI when controlling for age and sex (*β* = -2.57, *SE* = 0.80, *p* = 0.002). A mediation analysis indicated that while controlling for *APOE* ε4 status, BMI explained the association between fitness and plasma GFAP and NfL (Supplementary Fig. 1; indirect effects: *β* = 0.11, *SE* = 0.05 [95% CI 0.03 – 0.23]; *β* = 0.12, *SE* = 0.05 [95% CI 0.04 – 0.23], respectively).

## Discussion

We aimed to investigate if i) a 6-month exercise intervention influenced levels of plasma Aβ40, Aβ42, Aβ42/40 ratio, GFAP, NfL and p-tau181, ii) whether biomarker changes were related to changes in cognition after the exercise intervention, and iii) whether any findings differed by *APOE* ε4 carriage status. There was no effect of the exercise intervention on any plasma biomarker from pre- to post-intervention, and this did not vary dependent on *APOE* ε4 carrier status. At baseline, higher NfL was associated with poorer cognition. Exploratory analyses demonstrated that, at baseline, higher fitness was associated with higher GFAP and NfL levels in *APOE* ε4 non-carriers compared to ε4 carriers, an association which was explained by BMI.

Contrary to expectations, our exercise intervention had no significant effect on plasma blood biomarker levels. In cognitively unimpaired individuals, observational research shows an inverse association between self-reported physical activity and plasma NfL [20], Aβ42 and Aβ40 [31], which may depend on *APOE* ε4 carrier status [22]. However, there is very little research examining the effects of *exercise* (a structured or planned form of physical activity) on plasma biomarkers in cognitively unimpaired older adults. The distinction between exercise and physical activity is important because measurement of physical activity may capture other factors which contribute to reduced AD risk, such as social activities or community involvement, and these factors are less likely to be found in a structured exercise intervention (such as in the current study), potentially explaining these inconsistent findings. In other clinical populations, such as AD patients, exercise interventions have reduced plasma levels of p-tau and t-tau, but not NfL [23, 24], whereas in patients with multiple sclerosis, exercise interventions have reduced plasma levels of NfL and GFAP [32]. Since pathological processes likely differ between AD, multiple sclerosis and cognitively unimpaired individuals, further research is required in non-clinical populations.

2Our null findings for the effect of exercise on AD-related plasma biomarkers may be explained by the lack of mean change in biomarkers from pre- to post-intervention (Table 2). Biomarker levels are subject to intra-individual variability [33, 34], which may not be solely due to pathological processes, but rather external factors such as timing of sampling, or genuine biological variation (e.g., Aβ in cerebrospinal fluid (CSF) varies dependent on time of day [35]). Further research is needed to understand which factors influence such intra-individual variability, and may explain the lack of mean change in the current study. Further, plasma biomarkers are thought to reflect underlying brain processes, and 6-months may not be sufficient to induce Aβ-related brain changes which are subsequently reflected in blood. For example, plasma Aβ42/40 reflects levels of brain amyloid and may be sensitive to gray matter loss [5, 8]. However, a recent study found the maximum longitudinal change in plasma Aβ42/40 was ~ 1% per year in cognitively unimpaired older adults [36]. Thus, even using highly sensitive SIMOA technology, small changes over a relatively short time (e.g., 6 months) may be difficult to detect in blood. Longer exercise intervention periods and follow ups may more accurately reflect underlying AD-related disease processes, thus resulting in greater biomarker change.

Our baseline associations between plasma biomarkers and cognition are generally consistent with previous literature [14, 37]. However, some longitudinal studies in cognitively unimpaired older adults have found the strongest association between p-tau217 and cognition, followed by Aβ42/40 and/or GFAP, with smaller effect sizes observed for associations between NfL and cognition [38, 39]. In the current study, NfL was the biomarker most consistently associated with cognition, relating to performance across all cognitive domains except attention (Table 3). Disparate findings across studies may be owing to cohort differences since the two [38, 39] studies cited above were conducted in the BioFinder cohort, some of whom were recruited from memory clinics and thus likely have a greater proportion of ‘memory complainers’, and *APOE* ε4 carriers (~ 34% vs. 26%), than the current sample. Notably, one study in a cohort of similar age, ethnicity, and background to the current sample (the Australian Imaging, Biomarkers and Lifestyle, (AIBL) study cohort), used the same processing methodology and equipment as the current study, and showed results similar to ours [14]. Further, none of the above-mentioned studies have controlled for BMI in their analyses, and doing so strengthened the associations between NfL and cognition in the current study, as discussed below.

We found that in *APOE* ε4 carriers, higher baseline fitness was associated with lower baseline levels of GFAP and NfL. However, in ε4 non-carriers, higher baseline fitness was associated with higher baseline GFAP and NfL levels (Figs. 1 and 2). The inverse relationship between fitness, GFAP and NfL, especially the pattern in ε4 non-carriers, was unexpected given the general protective effects of physical activity (a large contributor to fitness) on AD risk [40], and cross-sectional associations between higher physical activity and lower NfL levels [20]. To better understand our results, we investigated differences between *APOE* ε4 carriers and non-carriers on cardiometabolic factors related to NfL, GFAP, and fitness. We found that in our cohort, BMI was significantly lower in ε4 carriers compared to non-carriers, and mediation analyses confirmed that BMI explained the association between fitness and NfL levels, and fitness and GFAP levels (Supplementary Fig. 1). These results indicate that it is not the effect of fitness per se*,* but rather its close association with lower BMI which likely explains these unexpected findings. There are two important conclusions the authors’ would like to highlight from these results, which are detailed below.

Firstly, from a methodological standpoint, future studies investigating blood biomarkers should consider BMI as a covariate in statistical models, especially if they are interested in biomarker differences between *APOE* ε4 carriers and non-carriers. Previous studies consistently demonstrate an inverse association between BMI and NfL, GFAP, and p-tau to a lesser extent [41–45]. One study showed that plasma NfL and GFAP levels increased after weight loss in morbidly obese patients, with biomarker sensitivity to total blood volume (as well as higher estimated glomerular filtration rate in obese individuals) as potential contributing factors [46]. The inverse association between BMI and plasma biomarkers is at least partially due to dilatation effects: people with lower BMI (and therefore lower total blood volume) have greater absolute levels of biomarkers in fractions of their plasma [45]. However, one study also found that BMI did not have a large confounding influence on plasma biomarkers in relation to biomarker levels in CSF [44]. These studies did not consider the relationship with *APOE,* and this avenue of research warrants further investigation.

Secondly, pathology-related explanations for associations between *APOE*, BMI and plasma biomarkers should be explored in future research. Higher BMI (≥ 25) in later life (specifically only in those > 70–75 years) is protective against cognitive decline and dementia, and unintentional weight loss is associated with prodromal AD in this age group; whereas higher BMI relates to increased AD risk in mid-life and early-late life (i.e., in those < 70 years) [47–49]. Those with a higher AD risk (i.e., ε4 carriers) show a more rapid decrease in BMI with ageing, which becomes evident at ~ 70 years old (the mean age of our sample) [50]. Thus, the current observed associations between *APOE*, BMI and plasma biomarkers may not be solely due to a confounding effect of BMI, but may also reflect other pathological processes associated with preclinical AD, since ε4 carriers are more likely on this trajectory. Indeed, Pichet Binette et al. [44] also concluded that the effects of BMI on plasma biomarkers may extend beyond a confounding effect (with the exception of NfL which remained associated with BMI after adjusting for brain Aβ). Unfortunately, the current study did not assess brain Aβ, but future research should investigate associations between BMI, *APOE* and plasma biomarkers in individuals with both high and low brain Aβ.

The current study was a well-controlled randomised clinical trial with a supervised exercise intervention, using ultrasensitive blood biomarker technologies and a comprehensive neuropsychological battery to measure cognition. However, our participant cohort was relatively homogenous: highly motivated, educated, and predominantly Caucasian, thus limiting the generalisability of our results. We also did not assess brain Aβ, a measure which may have improved our understanding of underlying pathological mechanisms. We were unable to collect blood samples at a later follow-up timepoint (beyond 6 months from baseline), which may have been more likely to detect sustained plasma biomarker changes. Finally, the control group in our exercise intervention received only one information session, and thus did not have the same involvement or social interaction (i.e., contact with the research staff) as those in the intervention groups, which may have influenced cognitive outcomes. Future research should aim to implement longer interventions and follow-up periods whilst considering how post-intervention exercise habits may influence biomarkers.

In sum, the current results show that an exercise intervention did not influence plasma levels of AD-related biomarkers. However, in our sample of cognitively unimpaired older adults, plasma biomarkers and cognition were cross-sectionally associated. We highlight that BMI is an important consideration when investigating plasma biomarkers, particularly when considering *APOE* ε4 status. Overall, blood biomarkers are a new, promising avenue for cost-effective, non-invasive markers of AD risk, and the current results contribute to an improved understanding of their best use in cognitively unimpaired older adults.

## Supplementary information

Below is the link to the electronic supplementary material.

Supplementary file1 (DOCX 30 KB)

## Data Availability

The datasets generated and/or analysed during the current study are not publicly available due to additional secondary analyses currently being conducted but are available from the corresponding author on reasonable request.
