# Supplementary material for: The impact of exercise on blood-based biomarkers of Alzheimer’s disease in cognitively unimpaired older adults
Source: GeroScience. 2024 Mar 15;46(6):5911–23. doi: 10.1007/s11357-024-01130-2 (PMC11493998; doi:10.1007/s11357-024-01130-2)
Supplement: Supplementary file 1 — Supplementary file1 (DOCX 30 KB) [file 11357_2024_1130_MOESM1_ESM.docx]

# Supplementary Material

| **Supplementary Table 1.** Cognitive assessments included in composite scores for each cognitive domain | |
| --- | --- |
| Cognitive domain | Included assessments |
| Global Cognition | - Digit Span - Cogstate one-back - Cogstate identification task - CVLT-II (learning, short delay recall, long delay recall, and recognition d`) - BVMT (learning and long delay recall) - Cogstate Groton Maze recall - Trail Making Test B - NIH-EXAMINER Phonemic fluency, Flanker, and Set-shifting tasks |
| Attention | - Digit Span (Forward only) - Cogstate identification task |
| Episodic Memory | - CVLT-II (short delay recall, long delay recall, and recognition d`), - BVMT long delay recall - Groton Maze recall |
| Executive Function | - Trail Making Test B - NIH-EXAMINER Phonemic fluency Flanker, and Set-shifting tasks |
| Abbreviations: CVLT-II, California Verbal Learning Test second edition; BVMT, Brief Visual Memory Test; NIH-EXAMINER, National Institutes of Health - Executive Abilities: Measures and Instruments for Neurobehavioral Evaluation and Research. | |

**-.41****

**-.30****

**-.28****

Supplementary Figure 1. Mediation for the effects of cardiorespiratory fitness on plasma GFAP and NfL levels through body mass index (BMI). Covariates include age, sex and *APOE* ε4 carrier status. Numbers represent partially standardised regression coefficients (all variables standardised except covariates).
* *p* <.05, ** *p* <.01, *** *p* <.001. Abbreviations: APOE, apolipoprotein E; GFAP, Glial Fibrillar Acidic Protein; NfL, Neurofilament Light chain.

| **Supplementary Table 2.** Linear models examining change in plasma biomarkers, change in cardiorespiratory fitness and change in cognition from pre- to post- exercise intervention | | | | | | |
| --- | --- | --- | --- | --- | --- | --- |
|  | Attention | Learning | Episodic Memory | Executive Function | Global Cognition | Cardiorespiratory fitness |
| Plasma Aβ40 | 0.03 (0.11) | -0.04 (0.11) | 0.01 (0.12) | 0.05 (0.12) | 0.01 (0.13) | 0.12 (0.11) |
| Plasma Aβ42 | -0.03 (0.11) | -0.18 (0.11) | 0.08 (0.11) | -0.04 (0.11) | -0.03 (0.11) | 0.10 (0.12) |
| Plasma Aβ42/40 | -0.08 (0.11) | -0.08 (0.10) | -0.15 (0.10) | 0.12 (0.11) | -0.07 (0.11) | -0.02 (0.12) |
| Plasma GFAP | -0.09 (0.12) | 0.01 (0.11) | -0.15 (0.11) | 0.00 (0.13) | 0.03 (0.12) | -0.08 (0.11) |
| Plasma NFL | -0.02 (0.12) | -0.01 (0.11) | -0.03 (0.11) | 0.11 (0.12) | -0.13 (0.12) | -0.00 (0.11) |
| Plasma p-tau181 | -0.03 (0.11) | 0.06 (0.11) | -0.09 (0.11) | -0.00 (0.12) | -0.17 (0.11) | 0.01 (0.12) |
| Change scores for every variable were used in these models. Cardiorespiratory fitness was treated as a predictor variable on plasma biomarker outcomes. Abbreviations: Aβ, amyloid-beta; GFAP, Glial Fibrillary Acidic Protein; NfL, Neurofilament Light chain; p-tau181, phosphorylated tau 181. Reported as standardised β (standard error). | | | | | | |
